# Genetic variation and mRNA expression of the *ELOVL6* and *CRTC2* genes in Kalmyk cattle

**DOI:** 10.1080/10495398.2025.2583795

**Published:** 2025-11-25

**Authors:** Nurlybay Kazhgaliyev, Kaster Nurgulsim, Aizhan Makhanbetova, Dulat Ibrayev, Kymbat Shaikenova, Zhandos Hasen, Saltanat Amantay, Saniya Zhumagaziyeva, Ilmira Mukhametzharova, Akkair Bakytzhan, Elmira Adilbekova

**Affiliations:** Faculty of Veterinary and Livestock Technology, S. Seifullin Kazakh Agro-Technical Research University (KATRU), Astana, Kazakhstan

**Keywords:** Kalmyk cattle, genes, SNPs, mRNA expression, MAS, association

## Abstract

Kalmyk cattle are an important meat breed in Kazakhstan, valued for their strong physique, genetic stability, and adaptability. In this study, we investigated the mRNA expression of ELOVL fatty acid elongase 6 (*ELOVL6*) and CREB-regulated transcription coactivator 2 (*CRTC2*) across multiple tissues, and further examined their genetic variations and associations with growth and carcass traits in 200 Kalmyk cattle. Expression analysis showed that *CRTC2* was most highly expressed in the heart and liver, whereas *ELOVL6* was predominantly expressed in the spleen and large intestine, highlighting their tissue-specific expression patterns. In addition, we identified a polymorphic SNP (g.16511290A > G) in the 3′UTR of *ELOVL6*, with three genotypes (AA, AG, GG) and the G allele being dominant (0.520). Polymorphism information content (PIC) analysis indicated high genetic diversity at this locus. Importantly, this SNP was significantly associated with live weight and body oblique length (*p* < 0.05), and individuals carrying heterozygous AG genotype showed higher body weight and length. Collectively, these findings suggest that g.16511290A > G within *ELOVL6* may serve as useful molecular markers for body measurements and meat traits, providing valuable resources for marker-assisted selection in beef cattle breeding programs.

## 1. Introduction

A key challenge that Kazakhstan’s agro-industrial sector must address in the coming years is the enhancement of high-quality beef production. One of the most promising strategies for tackling this issue lies in the development of specialized beef cattle breeding. Among the local breeds, Kalmyk cattle stand out as a popular meat breed in Kazakhstan due to their strong physique, genetic stability, and adaptability to harsh environments. The Kalmyk breed originated in the XVI-XVII centuries from cattle brought by Oirat tribes migrating from western Mongolia, and is now distributed mainly across southern Russia (Kalmykia, Rostov, Volgograd, Astrakhan) and parts of Kazakhstan.^1^ Previous studies have suggested that the Russian Kalmyk breed is genetically more similar to Mongolian cattle, with origins linked to Turano-Mongolian ancestry.^2^ According to Khamzina et al.^3^ the Kalmyk cattle population in Kazakhstan was estimated at approximately 23,000 individuals in 2022. Kalmyk cattle are distinguished by their origin, biological and productive qualities, as well as their body conformation type. The study on breeding stock of Kalmyk cattle in Kazakhstan indicate that calves are born with an average live weight of 22–26 kg and reach 165–175 kg by six months of age, with average daily gains of up to 1.0 kg in the first year of growth, reflecting good precocity.^4^ Despite their resilience and adaptability, the economic competitiveness of Kalmyk cattle compared to exotic commercial beef breeds is constrained by disadvantages such as lower intramuscular fat (marbling) and reduced subcutaneous fat deposition, which directly affect meat quality and consumer preference. Meanwhile, genomic characterization of Kalmyk cattle remains limited, and little is known about the genetic basis of their growth and carcass traits. Therefore, studying this breed provides valuable insights for both conservation and genetic improvement strategies.

Genetic studies play a crucial role in developing cattle breeds that produce meat with improved nutritional and quality profiles.^5^ In this regard, marker-assisted selection (MAS) offers a powerful tool, enabling breeders to identify and select animals carrying favourable genetic markers at an early stage, thereby reducing both the time and resources required to achieve targeted improvements.^6^ Among the various types of genetic variation, single nucleotide polymorphisms (SNPs) are the most prevalent and have become essential markers in livestock breeding. Understanding SNPs associated with key traits such as growth rate, fat deposition, and meat quality provides valuable insights for the design of efficient selection programs.^7^ The identification of favourable alleles can accelerate genetic progress by enhancing desirable traits, ultimately improving both the efficiency of beef production and the quality of the final product. In recent years, numerous SNPs linked to economically important traits have been identified across different cattle breeds.^8–10^ For example, polymorphisms in the calpastatin (*CAST*) gene have been significantly associated with meat tenderness in Nellore and Angus × Nellore cattle.^11^ Copy number variations (CNVs) in the *STAT5A* gene have been reported to influence growth traits in Chinese cattle.^12^

The ELOVL fatty acid elongase 6 (*ELOVL6*) and CREB-regulated transcription coactivator 2 (*CRTC2*) genes are particularly important for MAS studies targeting meat traits in cattle due to their significant roles in influencing growth, fat deposition, and meat quality. It has been reported that SNPs of the *ELOVL6* gene can influence the fatty acid composition of meat, affecting traits like marbling, fat deposition, and overall meat quality.^13^ The *ELOVL6* gene is involved in the elongation of long-chain fatty acids, which plays a vital role in lipid metabolism and it is particularly important for synthesizing monounsaturated fatty acids.^14^

The CREB-regulated transcriptional co-activators (*CRTC*) gene family consists of three members, including *CRTC1*, *CRTC2*, and *CRTC3* that also known as *TORC1*, *TORC2*, and *TORC3*. The *TORC2* gene plays a critical role in adipogenesis, lipogenesis, lipid esterification, and inhibition of lipolysis, and regulatory function in bovine adipocytes.^15^ It acts as a coactivator for transcription factors that drive the expression of genes involved in metabolism, therefore, plays a role in obesity.^16^ Previous study revealed that g.80G > T and g.93A > T SNPs of the *TORC1* gene were associated with superior body measurements in Qinchuan beef cattle.^17^ Moreover, SNPs in the *CRTC2* gene influence traits such as body weight gain, feed efficiency, and fat deposition in cattle.^18^ Khan et al.^19^ detected three novel SNPs (g.16534694G > A, g.16535011C > T, and g.16535044A > T) in the *TORC2* promoter that were associated with superior body measurements and carcass quality traits in Qinchuan cattle. This makes it a significant target for research in improving meat quality traits and yield in cattle.^20^

By identifying genetic markers that influence growth traits, it becomes possible to optimize meat quality, enhance growth performance, and promote overall sustainability in beef production. Therefore, the aim of this study was to analyze the expression patterns of the *ELOVL6* and *CRTC2* genes across different tissues, to screen for SNPs, and to evaluate their associations with body measurement traits in Kalmyk cattle.

## 2. Materials and methods

### 2.1. Sample collection and data acquisition

All experimental procedures involving animals were reviewed and approved by the Local Ethics Committee on Biological and Medical Ethics of S. Seifullin Kazakh Agrotechnical Research University (Protocol No. 1, dated 16 October 2024).

This research utilized blood samples from a total of 200 Kalmyk heifers aged 18–20 months. They were reared under uniform conditions at LLP ‘Qazaq Asyldary’ located in the Kobda district of the Aktobe region, Republic of Kazakhstan. The samples were transported to the laboratory and stored at −20 °C until further analysis. All cattle were subjected to a uniform feeding regimen and management practices on the farm.

In this study, 13 types of tissue samples (spleen, heart, liver, visceral fat, large intestine, right kidney, small intestine, duodenum, right lung, subcutaneous fat, skin, back muscle, and left gonad) were collected at the slaughterhouse of LLP ‘Qazaq Asyldary’ from a freshly slaughtered, clinically healthy 18-month-old Kalmyk bulls (*n* = 3). All samples were sterilized, placed in cryotubes and delivered to the laboratory in liquid nitrogen, after which they were stored in a freezer at −80 °C.

In addition, seven different morphometric indicators of the Kalmyk breed, including live weight (LW), body oblique length (BOL), height at the withers (HW), chest depth (ChD), chest girth (ChG), backfat thickness (BFT), and muscle thickness (MT) were recorded. Body measurements were determined using a tape measure and measuring stick, while carcass indicators were determined using ultrasonic devices.

### 2.2. RNA extraction, primer design and qRT-PCR

Total RNA was extracted from tissue samples using the RecoverAll^™^ Total Nucleic Acid Isolation Kit for FFPE (Thermo Fisher Scientific, Waltham, MA, USA) following the manufacturer’s protocol. Complementary DNA (cDNA) was synthesized from the extracted RNA using RevertAid First Strand cDNA Synthesis Kit (Thermo Fisher Scientific, Waltham, MA, USA). All procedures were performed according to the protocols provided with the kits.

Based on the mRNA sequences of the bovine *CRTC2* and *ELOVL6* genes (accession numbers: NM_001076250.1 and XM_015471503.3) published in the GenBank database, as well as the *β-actin* gene as an endogenous control (accession number: NM_173979.3), primers for qPCR were developed using NCBI Primer-BLAST software (Table 1).

**Table 1.** Primer sequence information for bovine *CRTC2* and *ELOVL6* genes.

Genes	Sequences	Location	Product size	Note
*CRTC2* (g.3001 C > T; g.3034 G > A; g.3467 T > C)	F: GGCGGTCTTGGAAGAGTTCA	Exon and intron	662 bp	SNPs
	R: CTCTGGCTCTCTCCTCCACT			
*ELOVL6* (g16379651A > G)	F1: CCATCATCTCTTCAGGGCGTG	Intron	308 bp	
	R1: AGGATACTGGGAGAAACGCA			
*ELOVL6* (g16458976A > G)	F2: TCTCTTTAGGGAAGGGGGCA	Intron	497 bp	
	R2: AGAGGAGATGGCCTGAGTGT			
***ELOVL6*** **(g16511290A > G)**	**F3:** **CTGCCCCACTATGCTGCAAT**	**3 ‘UTR**	**339 bp**	
	**R3:** **AGACAGTTAGAAGGATGGGAGTAAA**			
*β-actin*-F	CATCGGCAATGAGCGGTTCC	CDS	174 bp	mRNA
*β-actin*-R	CCGTGTTGGCGTAGAGGTC			
*CRTC2*-F	GAAGAATGGTGTCCCCGCTT		163 bp	
*CRTC2*-R	AGTGCAGATGGTAGTCGAAACA			
*ELOVL6*-F	TAGCACAGCCTCGGTCTAGT		114 bp	
*ELOVL6*-R	GGAGCTGCCCTTTCAAGAGT			

**Note:** F: forward; R: reverse; bold means polymorphism.

Gene expression levels were quantified by real-time PCR using PowerUp^™^ SYBR^™^ Green Master Mix (Thermo Fisher Scientific, Waltham, MA, USA) and specific primers (listed in Table 1) in a 20 μL reaction volume. The qRT-PCR procedure followed previously established conditions.^21^ The reactions were performed on a QuantStudio 7 Flex real-time PCR instrument (Thermo Fisher Scientific, Waltham, MA, USA). β-actin was used as the reference gene and relative expression levels of the target genes were calculated using the 2^^−ΔΔCt^ method. Statistical analysis was performed using GraphPad Prism version 10.0 (GraphPad Software, San Diego, CA, USA).

### 2.3. Genomic DNA isolation

DNA was extracted from blood samples using the GeneJET Genomic DNA Purification Kit (Thermo Fisher Scientific, Waltham, MA, USA) in accordance with the manufacturer’s protocol. The quality of the DNA obtained was analyzed using agarose gel electrophoresis and spectrophotometry. Then samples that met the required concentration were diluted to 50 ng/μl and stored at −40 °C.

### 2.4. Primer design and genotyping

For the design of primers, the sequence of the bovine *CRTC2* and *ELOVL6* genes were referenced from NCBI (Accession numbers: NC_037330.1 and NC_037333.1), while the primers targeting SNPs g16379651A > G; g16458976A > G; g16511290A > G^22^ in the *ELOVL6* gene and g.3001 C > T, g.3034 G > A, g.33467 T > C^18^ in *CRTC2* gene were created using NCBI Primer-BLAST (see Table 1).

Polymerase chain reaction (PCR), agarose gel electrophoresis and Sanger sequencing were employed to identify polymorphisms. Each PCR reaction was carried out in a total volume of 25 µL. The reaction mixture contained 12.5 µL of DreamTaq PCR Master Mix (2X) (Thermo Fisher Scientific, Waltham, MA, USA), 1 µL of forward primer, 1 µL of reverse primer, 3 µL of template DNA, and 7.5 µL of ddH2O. The PCR reaction condition is given in Table 2. The resulting PCR products were subjected to fractionation on a 3.5% agarose gel, and subsequently sent to sequencing. All individuals underwent individual genotyping.

**Table 2.** PCR reaction conditions.

Step	Temperature, °C	Time	Number of cycles
Initial denaturation	95.0	3 min	1
Denaturation	95.0	30 s	35
Annealing	63.0	30 s	
Extension*	72.0	1 min	
Final extension	72.0	5 min	1

### 2.5. Statistical and bioinformatical analyses

Population genetics parameters were evaluated with the GDIcall Online Calculator (available at http://www.msrcall.com/Gdicall.aspx). The Hardy-Weinberg equilibrium (HWE) was assessed using the SHEsis program (accessible at http://analysis.bio-x.cn). Statistical analysis was conducted using general linear model (GLM) in SPSS Statistics version 25.0 (IBM Corp., Armonk, NY, USA), to analyze the significance of associations between genotypes and body morphometric characteristics in Kalmyk cattle, with a *P* < 0.05 deemed statistically significant.

The influence of genotypes on growth traits was examined using the following statistical model: Y*_ijk_* = μ + A*_i_* + G*_j_* + E*_ijk_*, where Y*_ij_* represents the body measurement trait, μ denotes the mean for each trait, A*_i_* indicates the fixed effect of genotype, G*_j_* refers to the effect of age, and E*_ijk_* signifies random error.^23^

The correlation between phenotypic traits was analyzed by constructing a diagram in the RStudio software (PBC, Boston, MA, USA).

## 3. Results

### 3.1. mRNA expression analysis of the *CRTC2* and *ELOVL6* genes

**Figure 1.**
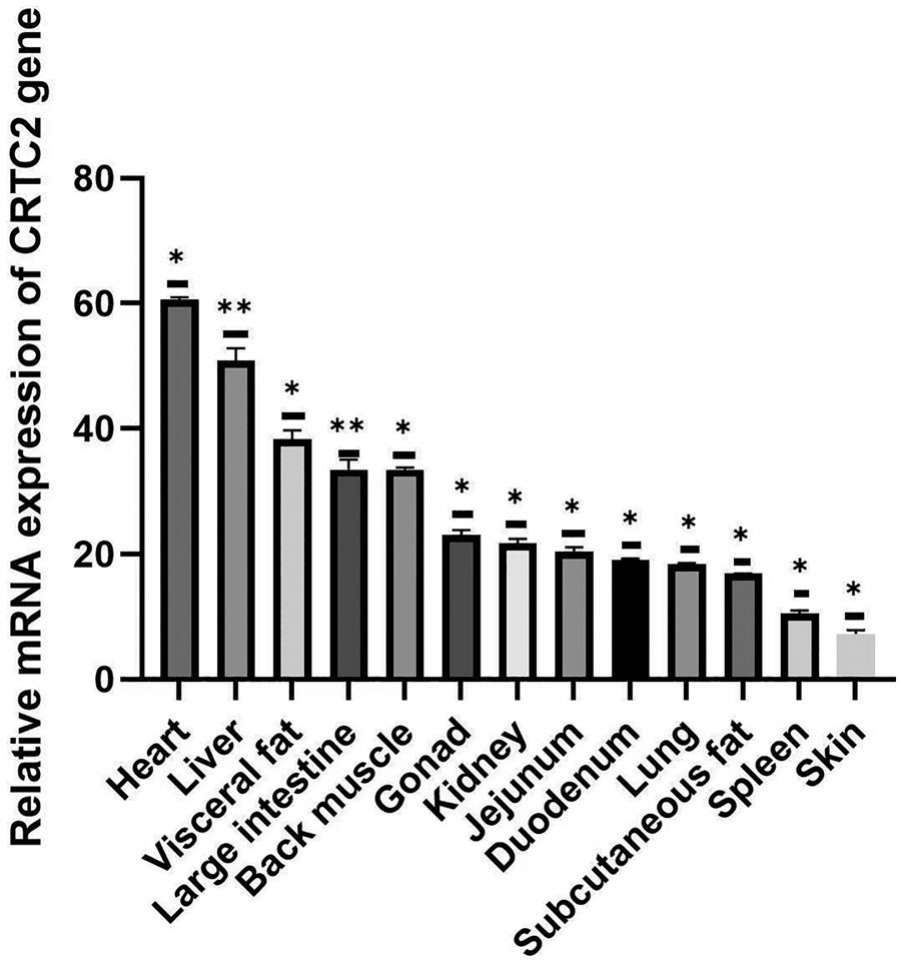
Expression analysis of bovine *CRTC2* gene in different tissues.

We analyzed the mRNA expression patterns of the *CRTC2* and *ELOVL6* genes in various tissues of Kalmyk cattle to investigate their tissue-specific expression profiles and potential biological roles. The mRNA expression of the *CRTC2* gene (Figure 1) was highest in the heart (*p* < 0.05), followed by the liver (*p* < 0.01) and visceral fat (*p* < 0.05). Moderate expression levels were detected in the large intestine, back muscle, gonad, and kidney, all showing significant differences *(p* < 0.05). Low expression was observed in the jejunum, duodenum, lung, subcutaneous fat, spleen, and skin, also with significant differences (*p* < 0.05). The highest transcriptional activity observed in the heart and liver, may indicate its potential role in the regulation of energy metabolism and glucose metabolism.

**Figure 2.**
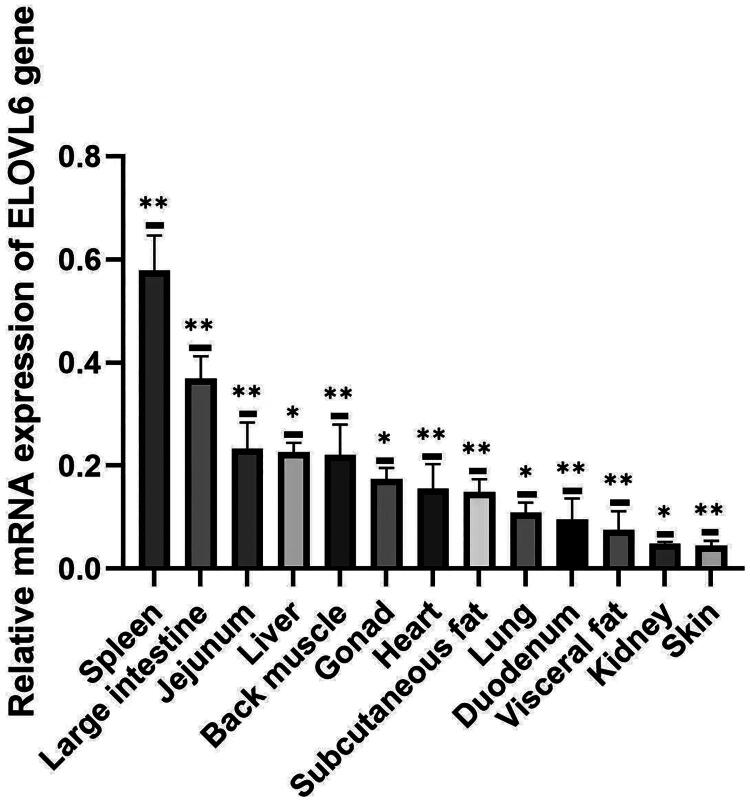
Expression analysis of bovine *ELOVL6* gene in different tissues.

Figure 2 shows the relative expression of *ELOVL6* mRNA in various tissues of Kalmyk cattle. According to the analysis results, the highest level of *ELOVL6* mRNA expression was observed in the spleen and large intestine (*p* < 0.01). Medium expression levels were recorded in the small intestine, liver, spinal muscle, gonads, subcutaneous adipose tissue, and cardiac tissue (*p* < 0.05). The lowest expression levels were observed in the lung, duodenum, visceral fat, kidney, and skin tissue (*p* < 0.05). The results obtained indicate the tissue-specific nature of *ELOVL6* gene expression in the tissues of Kalmyk cows. This gene is actively transcribed in the spleen and large intestine, which may indicate its role in lipid metabolism and energy exchange in these tissues.

### 3.2. Correlation analysis of body measurement traits

**Figure 3.**
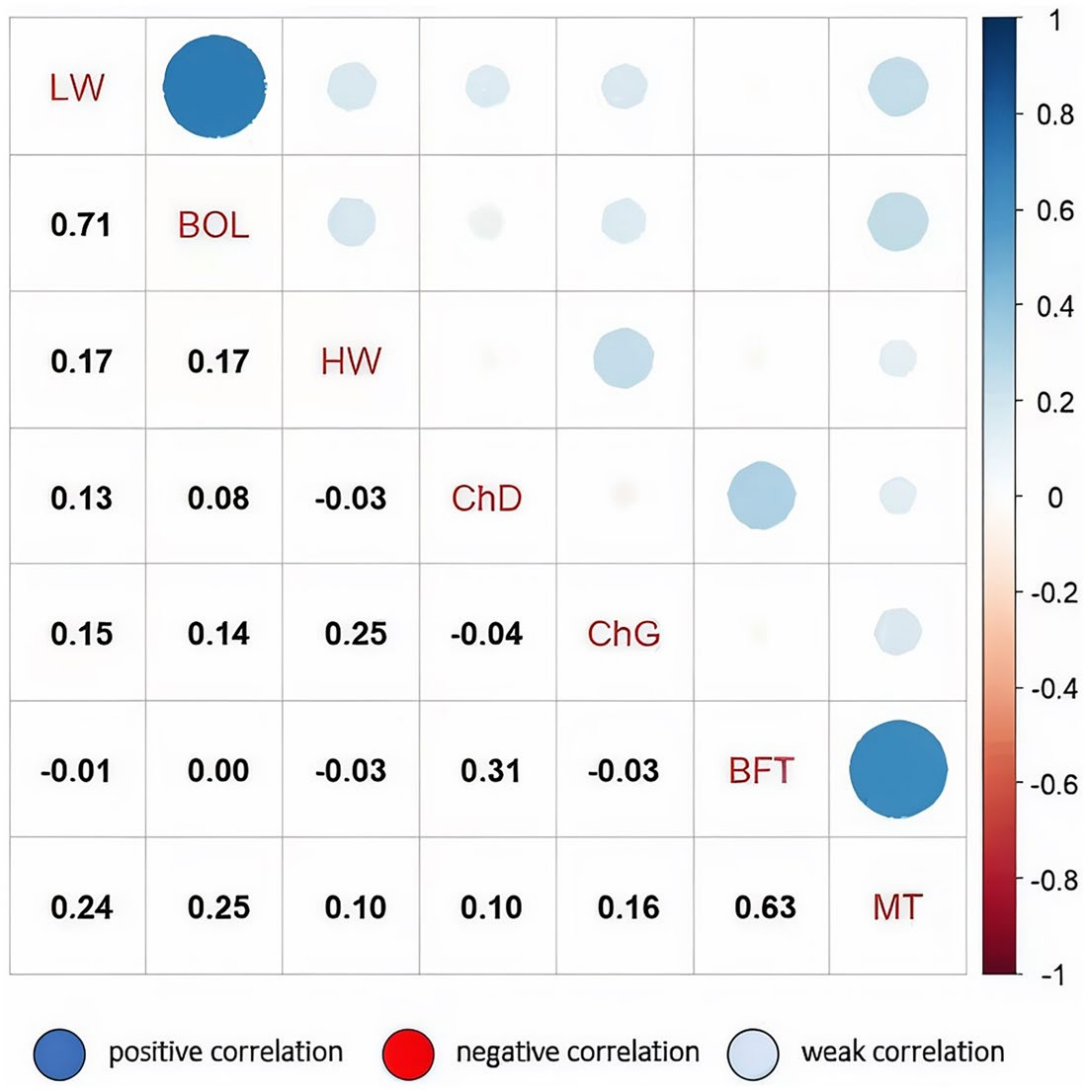
Correlation diagram for growth traits. LW, live weight, BOL, body oblique length, HW, height at the withers, ChD, chest depth, ChG, chest girth, BFT, backfat thickness, MT, muscle thickness. The size of the circles reflects the absolute value of the correlation coefficient, while the color represents the direction and magnitude of the correlation (blue = positive, red = negative). Correlation values range from −1 (perfect negative) to +1 (perfect positive).

To examine the phenotypic relationships among these traits, we conducted correlation analysis (Figure 3). A strong positive correlation was observed between LW and BOL (r = 0.71), suggesting that heavier animals tend to have longer body frames. Similarly, MT was strongly associated with BFT (r = 0.63), indicating parallel development of muscle and fat deposition. A moderate correlation was detected between BFT and ChD (r = 0.31), while weak correlations were observed between HW and ChG (r = 0.25), reflecting that animals with greater frame size tended to have slightly broader chest dimensions. In contrast, most other trait pairs showed weak or negligible correlations (r values between −0.05 and 0.25), suggesting little phenotypic association. Overall, these results highlight that growth traits are interconnected, with body size and carcass composition traits developing in parallel, thereby providing a foundation for subsequent genetic association analysis.

### 3.3. Identification of genetic variations and genetic parameters

**Figure 4.**
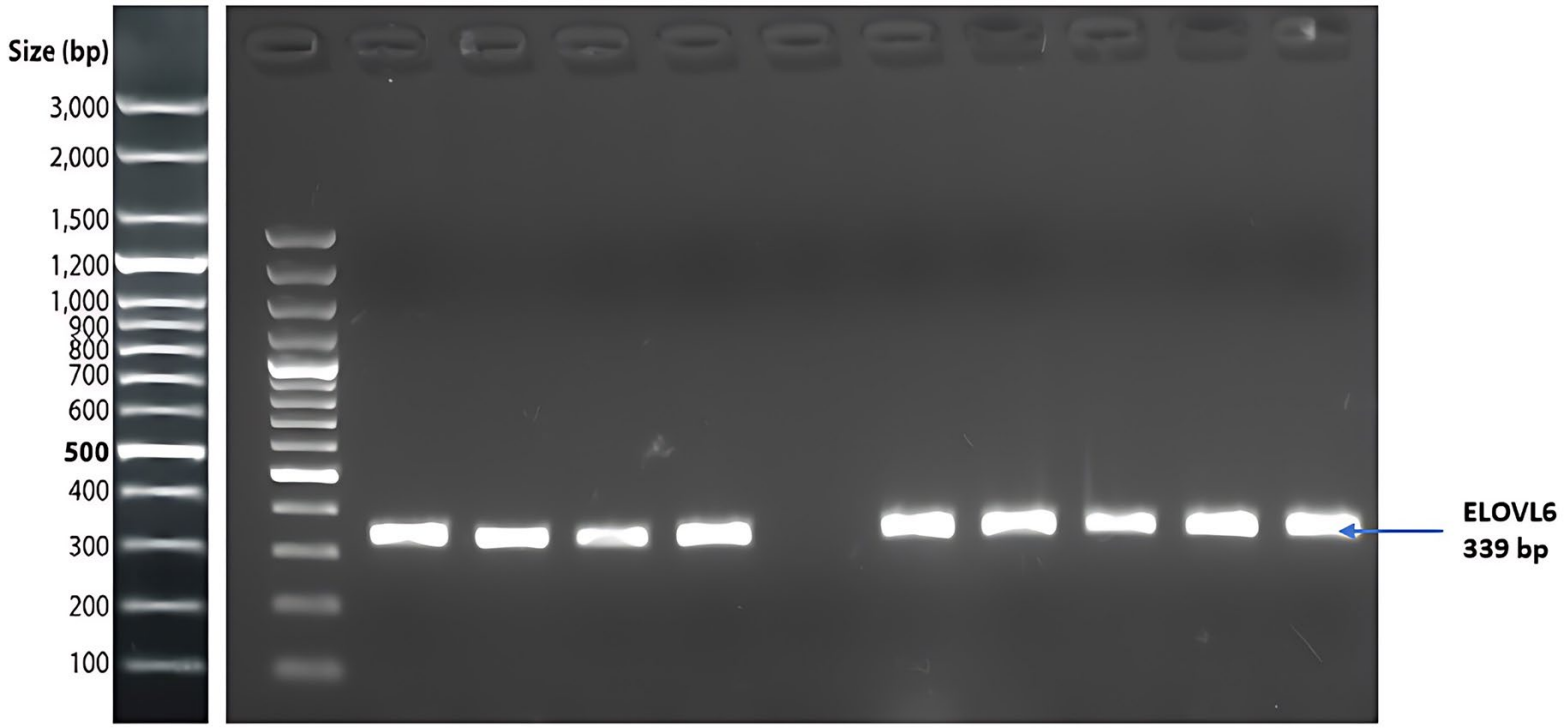
Electrophoresis of ELOVL6-g.16511290A > G locus.

**Figure 5.**
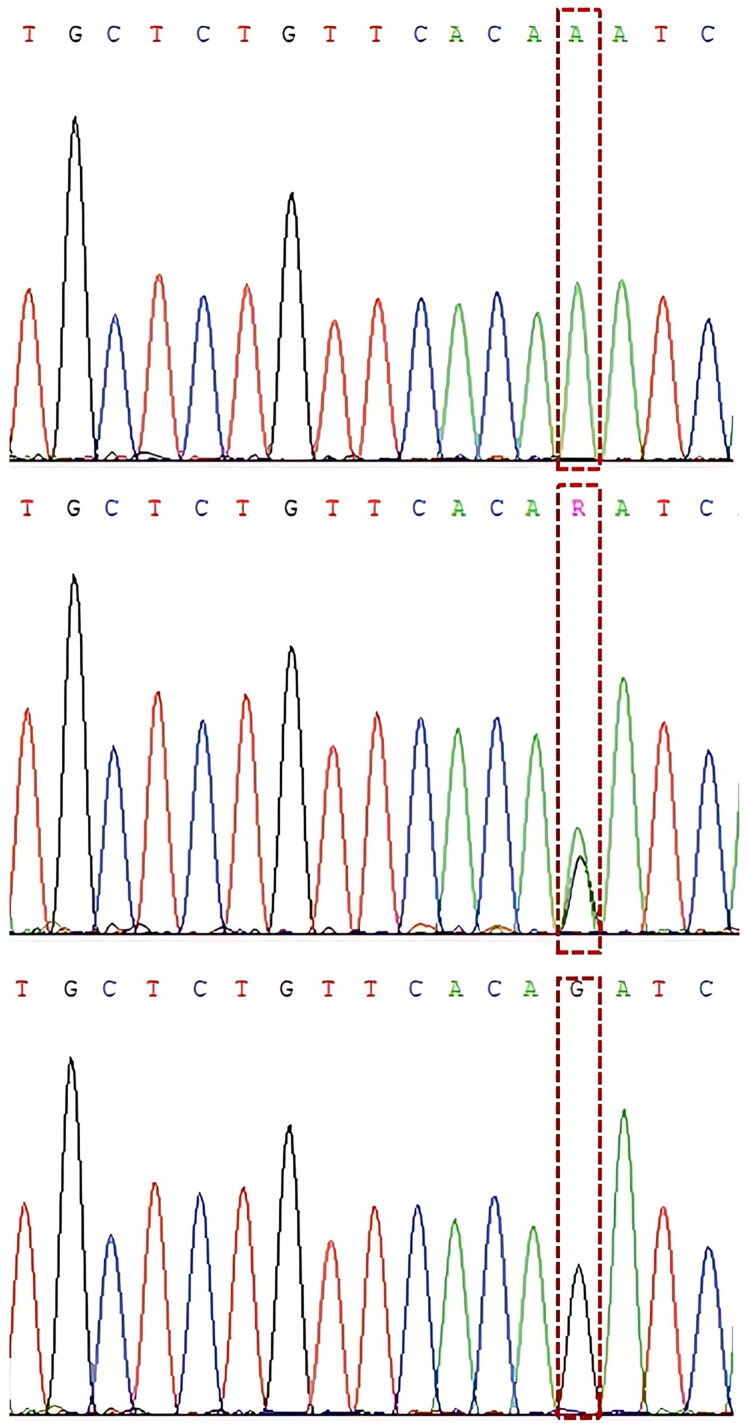
Sequencing results of the ELOVL6-g.16511290A > G locus.

The sequencing results revealed no polymorphisms in the *CRTC2* gene in Kalmyk cattle. In contrast, analysis of the *ELOVL6* gene revealed a single polymorphic site (g.16511290A > G) located in the 3′UTR region. The gel electrophoresis results are presented in Figure 4. The sequencing data further confirmed the presence of three genotypes, namely AA, AG, and GG, at this locus (Figure 5).

The genetic parameters, including genotypic frequency, allelic frequency, homozygosity, heterozygosity, effective allele numbers, polymorphism information content of the SNP in the *ELOVL6* gene in the Kalmyk cattle are shown in Table 3. For g16511290A > G locus, the GG genotype as present at a higher frequency (0.370) than the AA (0.325) and AG (0.305) genotypes, the frequency of the A allele was 0.477, and the G allele was the dominant allele (0.523). SNPs showed a high genetic diversity (0.375). In addition, this marker showed a deviation from Hardy-Weinberg equilibrium (HWE) in the tested sample (*P* = 0.006).

**Table 3.** Population genetic indices of loci in the Kalmyk breed.

Loci	Frequencies					*Ho*	*He*	*Ne*	HWE *P*-Value	PIC
	Genotypes			Alleles						
*ELOVL6* (g16511290A > G)	AA 0.325	AG 0.305	GG 0.370	A 0.477	G 0.523	0.501	0.499	1.996	*P* = 0.006	0.375

**Note:** *Ho*, homozygosity; *He*, heterozygosity; *Ne*, effective allele numbers; PIC, polymorphism information content; HWE, Hardy-Weinberg equilibrium.

### 3.4. Association of SNP with body measurement traits in Kalmyk cattle

Furthermore, we examined the association of *ELOVL2* polymorphism with growth traits (Table 4). The GLM analysis revealed a significant effect of genotype on live weight (*p* < 0.001). The effect size (Partial η^2^ = 0.121) indicates that approximately 12.1% of the variance in body weight can be explained by differences among genotypes, which represents a moderate effect. Animals with the AG genotype had the highest mean body weight (351.15 ± 10.65 kg) compared to the AA and GG genotypes (Table 4). Similarly, a significant association between genotypes of g16511290A > G and BOL (*p* = 0.004). Individuals with genotype AG showed the highest mean BOL (141.62 ± 6.84). Genotype accounted for approximately 5.5% of the variance in BOL, suggesting a small but statistically significant effect.

**Table 4.** The association analysis between growth traits and the SNP g16511290A > G of the *ELOVL2* gene.

Growth traits	Observed genotypes (Mean ± SD)			F-Value	*P*-values	Partial η^2^
	AA	AG	GG			
LW, kg	338.14 ± 17.2^A^	351.15 ± 10.6^B^	340.74 ± 15.43^A^	**13.572**	**0.000003**	**0.121**
BOL, cm	137.48 ± 6.49^a^	141.62 ± 6.84^b^	139.03 ± 7.35^ab^	**5.746**	**0.004**	**0.055**
HW, cm	120.34 ± 2.3	120.62 ± 1.6	121.08 ± 3.4	1.419	0.244	0.014
ChD, cm	56.01 ± 4.7	57.41 ± 4.6	56.95 ± 3.6	1.729	0.180	0.017
ChG, cm	124.40 ± 4.9	124.26 ± 6	123.08 ± 6.5	1.066	0.347	0.011
BFT, cm	0.74 ± 0.1	0.71 ± 0.1	0.72 ± 0.1	1.017	0.364	0.010
MT, cm	3.44 ± 0.1	3.42 ± 0.1	3.42 ± 0.1	0.775	0.462	0.008

**Note:** LW: live weigh; BOL: body oblique length; HW: height at the withers; ChD: chest depth; ChG chest girth; BFT: backfat thickness, MT: muscle thickness, η^2^: effect size. Different letters (A, B) indicate a significant difference a*t p* < 0.001, while lowercase letters (a, b) indicate a significant difference at *p* < 0.05.

No significant differences were observed among genotypes for height at withers, chest depth, chest girth, backfat and muscle thickness (*P* > 0.05), suggesting that the effect of the g16511290A > G polymorphism is specific to live weight and body oblique length rather than general body conformation traits.

## 4. Discussion

The Kalmyk cattle is a unique source of genetic material, which has been used to create modern meat cattle breeds such as the Kazakh white-headed cattle.^3^ MAS is a powerful tool in livestock breeding that accelerates the development of improved varieties by using molecular markers linked to desirable traits. Understanding gene function is crucial when selecting genes for MAS because it enables breeders to target traits that significantly improve economically important traits.^24^

In the present study, *ELOVL6* showed predominant expression in the spleen and large intestine, which may reflect its function in fatty acid elongation and immune–metabolic interactions, as the spleen is central to immune regulation and the intestine plays a critical role in nutrient absorption and lipid metabolism. Plenty studies have been proposed that *ELOVL6* plays a significant role in energy metabolism and insulin sensitivity (Matsuzaka, 2020)^25^. In the study by Junjvlieke et al.^21^^,^^26^ *ELOVL6* has been studied for its potential impact on the fatty acid composition in bovine adipocytes. Transcriptome analysis of adipose tissue revealed that miR-211 and miR-331-5p showed an inverse relationship with the elongation of *ELOVL6.*^26^ It was observed that *ELOVL6* knockout mice exhibited weight gain, increased subcutaneous white adipose tissue mass, and impaired carbohydrate metabolism.^27^

In this study, we observed one polymorphic SNP (g.16511290A > G) in the *ELOVL6* gene, and the observed deviation from Hardy–Weinberg equilibrium at this locus may be attributed to selection pressure, small effective population size, or potential sampling effects influencing the allele distribution in the studied population. Furthermore, the association study results exhibited that g.16511290A > G variation in the *ELOVL6* gene was significantly related to live weight and body oblique length in Kalmyk cattle, which is mainly attributed to its function. To the best of our knowledge, this is first on mRNA expression and genetic variations in this gene and their association with body measurement traits in Kalmyk cattle.

Previously, SNPs in this gene were contributed to somatic cell score and milk fat content in cattle.^22^ More recently, it was found that variation in *ELOVL6* was correlated with carcass composition and meat quality in pigs.^28^ In sheep, RNA-sequencing analysis has identified *ELOVL6* as a gene involved in lipid metabolism and fat deposition.^29–31^ This gene was identified as a significant locus in the genome-wide association study, which plays a role in the genetic regulation of fatty acid composition in pigs.^32^ Study on *ELOVL6* in milk composition revealed that miR-206 hinders milk fatty acid production and lipid buildup by targeting *ELOVL6* in bovine mammary epithelial cells.^33^ Therefore, variations in this gene may lead to changes in the fatty acid profile in tissues, potentially influencing body composition and fat distribution in cattle.

The tissue-specific expression analysis demonstrated that *CRTC2* was most highly expressed in the heart and liver, suggesting a potential role in regulating energy metabolism and cardiac function, consistent with its known involvement in gluconeogenesis and stress response pathways.^16^^,^^34^ In obese mice, hepatic overexpression of an mTOR-defective CRTC2 mutant improved lipogenesis and insulin sensitivity, highlighting the role of CRTC2 in mTOR-dependent lipid regulation in fed and obese states.^16^ It was reported that *CRTC2* (also known as *TORC2*) expression was greatest in the abomasum in Qinchuan cattle.^35^ siRNA-mediated knockdown of the *TORC2* gene followed by RNA sequencing revealed that its downregulation impairs bovine adipocyte differentiation.^36^

The absence of polymorphisms in the *CRTC2* gene observed in Kalmyk cattle may reflect breed-specific genetic background, strong purifying selection, or a relatively small effective population size, in contrast to reports of variation in other breeds. Previously, observed that SNPs in *CRTC2* were associated with several growth and meat traits in cattle. *CRTC1* gene acts as a coactivator for the transcription factor CREB (cAMP response element-binding protein), which is important in metabolism and skeletal muscle disorders.^34^ For instance, previous study identified the core promoter region of the *TORC1* gene and it is regulated via NRF1 and Smad3 transcription factors, providing insights for improving intramuscular fat in cattle.^37^ It was demonstrated that bta-miR-149-5p directly targets *CRTCs*, specifically *CRTC1* and *CRTC2* and plays a role in regulating adipogenesis.^38^ Also, *CRTC2* interacts with AMP-activated protein kinase (AMPK), which is a critical energy sensor in cells. Activation of AMPK can promote fatty acid oxidation and suppress lipogenesis, influencing body composition and fat deposition.^39^ Notably, the expression levels of *CRTC2* in the mouse mammary gland were significantly elevated during lactation compared to the dry and puberty stages, while the phosphorylation of *CRTC2* at Ser171 remained unchanged, which further reinforces the idea that *CRTC2* plays a crucial role as a transcriptional coactivator in the process of milk fat synthesis via mechanistic target of rapamycin (mTOR).^40^ The mTOR pathway is essential for regulating cell growth and metabolism in response to nutrients and growth factors.^41^ Hence, *CRTC1* gene may interact with these pathways to influence muscle growth and fat deposition.

Furthermore, the identified SNP (g.16511290A > G) was located in the 3′UTR region of the *ELOVL6* gene, a regulatory region that can influence mRNA stability, localization, and translation efficiency, thereby potentially affecting gene expression and growth traits. Specifically, the SNP could create or disrupt microRNA (miRNA) seed sites or RNA-binding-protein (RBP) motifs, thereby changing mRNA decay rates and translation efficiency.^42^ It may also change the 3′UTR structure or polyadenylation, which can affect mRNA stability and translation.^43^ For instance, the SNP in the 3′UTR of NCF4 lies within the bta-miR-2426 binding site, and cows carrying the GG genotype exhibited higher NCF4 mRNA expression than those with the AA genotype, suggesting regulation through a miRNA–mRNA interaction.^44^ Coding variations or loci in regulatory regions in genes play a crucial role in determining phenotypic traits in livestock, influencing characteristics such as growth rate, milk production, and disease resistance.^45^ By studying these variations, we can gain insights into the biological mechanisms that underpin phenotypic diversity in livestock, leading to innovations in animal husbandry practices. Together, these findings contribute to the molecular characterization of fat metabolism related genes in Kalmyk cattle and provide candidate genetic markers for further evaluation in breeding programs.

## Conclusion

This study provides novel insights into the functional roles and genetic variation of the *CRTC2* and *ELOVL6* genes in Kalmyk cattle. The tissue-specific expression patterns, with *CRTC2* highly expressed in the heart and liver and *ELOVL6* predominantly in the spleen and large intestine, suggest their involvement in metabolic and growth-related processes. Moreover, the identification of a polymorphic SNP (g.16511290A > G) in the 3′UTR of *ELOVL6*, which was significantly associated with live weight and body oblique length, highlights its potential as a molecular marker. Collectively, these findings indicate that the SNP g.16511290A > G in *ELOVL6* may serve as useful marker for marker-assisted selection aimed at improving body measurements and meat production traits in beef cattle.

## Supplementary Material

Original Image for Figure 4.jpg
