# Supplementary figures and images for: Genetic variation and mRNA expression of the ELOVL6 and CRTC2 genes in Kalmyk cattle
Source: Anim Biotechnol. 2025 Nov 25;36(1):2583795. doi: 10.1080/10495398.2025.2583795 (PMC12875334; doi:10.1080/10495398.2025.2583795)

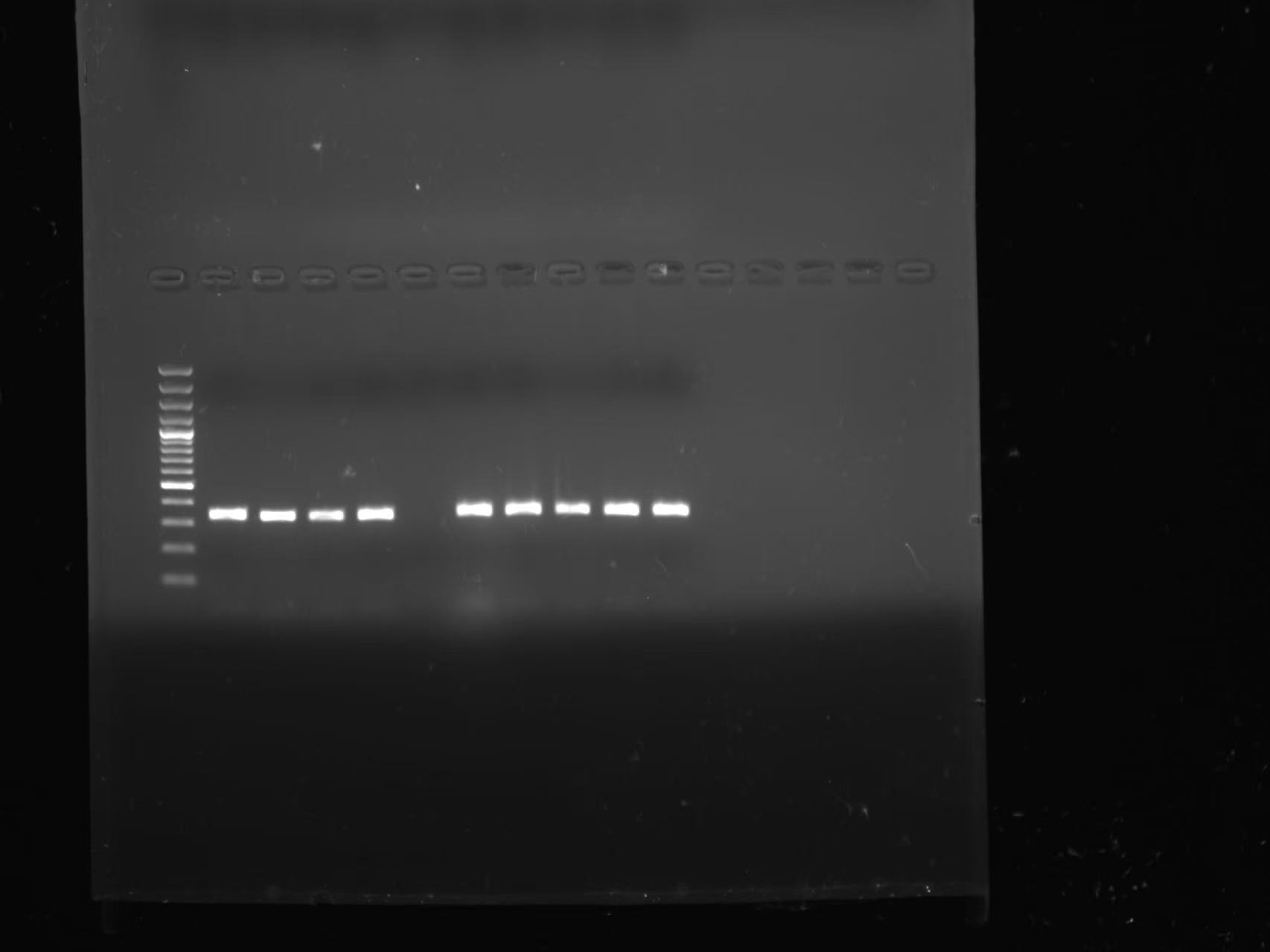

Supplement: Original Image for Figure 4.jpg [file LABT_A_2583795_SM2642.jpg]
